# Rattles of the Rattle-Snake

**Published:** 1874-01

**Authors:** 


THE RATTLES OF THE RATTLESNAKE.—The formation of rattles upon the tail of a rattlesnake is a curious phenomenon. The notion that one is developed each year is incorrect. Young ones have been known to have six or more; sometimes two or three appear in a single year. The number seldom exceeds fifteen. The skin of one that was six feet long, in the Museum of the Long Island Historical Society of Brooklyn, has fourteen rattles. De Kay cited, in 1842, the *Clarion* newspaper, published at Bolton, New York, which stated that two men killed, in three days, in the town of Bolton, at Lake George, 1,104 rattlesnakes, some of which carried fifteen to twenty rattles. They were killed for their oil. The same author states, on the authority of the *Columbian Magazine* for November, 1786, that a rattlesnake was killed, having forty-four rattles, which seems an incredible number. The use of the rattles is a subject of discussion. They are evidently well developed—not rudimental merely—and the conclusion is irresistible that they are of service to the creature. We cannot suppose that organs which are constant in a class of animals, could have originated, if entirely useless and unserviceable to it. Prof. Aughey suggests that the whirring rattle is a call-note by the animal to its mate. That it was thus used on one occasion he was an eye-witness. Again, it may be used to terrify its enemies; or to paralyze its victims with fright, or to call assistance in danger. He says: "I once witnessed an attack by seven hogs on a rattlesnake. Immediately the snake rattled, and three others appeared; but the hogs were victorious."—*Popular Science Monthly for January.*

